# Including eye care in the Integrated Management of Childhood Illness (IMCI) programme in Bangladesh

**Published:** 2022-03-01

**Authors:** AHM Enayet Hussain, Khaleda Islam

**Affiliations:** 1 Director General: Directorate of Medical Education, Ministry of Health and Family Welfare, Bangladesh.; 2 Director Primary Health Care (Retired) & Directorate General of Health Services: Mohakhali, Dhaka, Bangladesh.

**Bangladesh has successfully integrated primary eye health care for children into the country's Integrated Management of Childhood Illness (IMCI) programme.**

**Figure:**
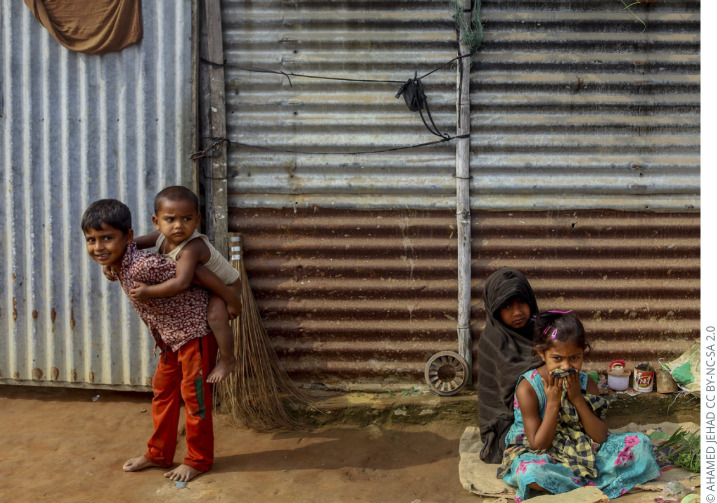
Children play outside. **BANGLADESH**

In Bangladesh, primary eye health care (PEHC) for all ages was included in the National Eye Care Operational Plan. However, children were not specifically being reached, as eye care was not included in the World Health Organization's (WHO's) Integrated Management of Childhood Illness (IMCI) programme. To address this, in 2016, with support from WHO, an initial package of interventions to identify and refer children with eye conditions by primary health care workers was developed and pilot tested in one sub-district. As this initiative was successful, it was decided to advocate that eye care be included in the national IMCI program to make it sustainable.

The National Newborn Health and IMCI programme of the ministry of health and family welfare took the initiative. Several consultation meetings were held in 2017, involving stakeholders in newborn care, child health, and eye care from the government, United Nations agencies, non-governmental organisations, professional associations, and district program managers. It was estimated that including eye care and screening in IMCI would benefit around 7.5 million children every year, using the same resources and workforce with some additional training. In May 2018, government policymakers, WHO, UNICEF, and technical experts agreed to include eye care in IMCI, and Sightsavers supported a pilot project in one district.

A technical group developed the curriculum and training materials, which included screening using a torch. Guidelines for identifying eye problems were produced, and strong referral mechanisms to the district hospital eye department were developed. IMCI recording forms for children aged <2 months and 2–59 months were updated to include eye conditions, and referral slips and reporting forms were designed. The eye conditions screened for in children were a white pupillary reflex, watering, red or discharging eye(s), and structural abnormalities. Eye injuries, squints, and concerns about vision were added for older children. Community health workers attached to facilities delivering IMCI were also requested to promote awareness about eye conditions in children in the community.

IMCI staff members started screening children in July 2018, and referring cases requiring further eye care to the sub-district medical officer for treatment or referral to the district hospital ophthalmologist. The technical group members conducted regular monitoring and supportive supervision to monitor the quality of care and reporting. The IMCI national database (District Health Information Software 2 [DHIS2] of the Director General of Health Services) was modified to include eye conditions in children, and health facilities used this system for monthly reporting.

Based on the lessons learned from the pilot project, the ministry of health and family welfare included the eye care component of the IMCI protocol in the National Newborn Health and IMCI programme and scaled it up nationwide. A budget was allocated in the National Newborn Health operational plan to train IMCI staff. National data are being recorded in the updated DHIS2 platform, and the National Newborn Health programme monitors and provides supportive supervision. All these initiatives led to an increasing number of children benefitting from eye care services.

## Lessons learned

IMCI staff could screen and refer cases confidently after basic training. Additional in-service training increased performance.

The eye care component of the IMCI was included in monthly facility coordination meetings, which increased awareness among all stakeholders.

Health promotion by community health workers increased awareness of eye conditions and referrals from the community to primary health care clinics.

Good follow-up, coordination, and strong referral mechanisms improved the quality of eye care.

Including IMCI (which now has the eye care component) in the curriculum of medical, nursing, and paramedical students, increases effectiveness.

## Conclusions and recommendations

Integrating eye care into the IMCI program is a feasible, efficient, effective and sustainable way to provide primary eye health care for children in Bangladesh. Basic equipment, logistical support and training, with refresher training as trained staff may be transferred, are essential. Incorporating eye care into IMCI is an excellent example of enhancing available resources to address avoidable blindness in children by strengthening the health system to ensure universal eye care in Bangladesh.

